# Treatment of COVID-19 with remdesivir in the absence of humoral immunity: a case report

**DOI:** 10.1038/s41467-020-19761-2

**Published:** 2020-12-14

**Authors:** Matthew S. Buckland, James B. Galloway, Caoimhe Nic Fhogartaigh, Luke Meredith, Nicholas M. Provine, Stuart Bloor, Ane Ogbe, Wioleta M. Zelek, Anna Smielewska, Anna Yakovleva, Tiffeney Mann, Laura Bergamaschi, Lorinda Turner, Frederica Mescia, Erik J. M. Toonen, Carl-Philipp Hackstein, Hossain Delowar Akther, Vinicius Adriano Vieira, Lourdes Ceron-Gutierrez, Jimstan Periselneris, Sorena Kiani-Alikhan, Sofia Grigoriadou, Devan Vaghela, Sara E. Lear, M. Estée Török, William L. Hamilton, Joanne Stockton, Josh Quick, Peter Nelson, Michael Hunter, Tanya I. Coulter, Lisa Devlin, CITIID-NIHR COVID-19 BioResource Collaboration, MRC-Toxicology Unit COVID-19 Consortium, John R. Bradley, Kenneth G. C. Smith, Willem H. Ouwehand, Lise Estcourt, Heli Harvala, David J. Roberts, Ian B. Wilkinson, Nick Screaton, Nicholas Loman, Rainer Doffinger, Paul A. Lyons, B. Paul Morgan, Ian G. Goodfellow, Paul Klenerman, Paul J. Lehner, Nicholas J. Matheson, James E. D. Thaventhiran

**Affiliations:** 1 Department of Clinical Immunology, Barts Health, London, UK; 2 UCL GOSH Institute of Child Health Division of Infection and Immunity, Section of Cellular and Molecular Immunology, London, UK; 3 Centre for Rheumatic Diseases, King’s College London, London, UK; 4 Department of Infection, Barts Health NHS Trust, London, UK; 5 Department of Pathology, University of Cambridge, Addenbrooke’s Hospital, Cambridge, UK; 6 Peter Medawar Building for Pathogen Research, South Parks Rd, Oxford, OX1 3SY UK; 7 Nuffield Department of Clinical Medicine, University of Oxford, Oxford, UK; 8 Cambridge Institute of Therapeutic Immunology and Infectious Disease, Jeffrey Cheah Biomedical Centre, Cambridge Biomedical Campus, Cambridge, UK; 9 Department of Medicine, University of Cambridge School of Clinical Medicine, Cambridge Biomedical Campus, Cambridge, UK; 10 Systems Immunity Institute and Dementia Research Institute, Cardiff University, Cardiff, UK; 11 Division of Virology, Department of Pathology, University of Cambridge, Addenbrookes Hospital, Cambridge, UK; 12 PHE – Public Health England Laboratory, Cambridge. Box 236, Cambridge University Hospitals NHS Foundation Trust, Hills Road, Cambridge, UK; 13 Medical Research Council Toxicology Unit, University of Cambridge, Gleeson Building, Tennis Court Road, Cambridge, CB2 1QW UK; 14 R&D Department, Hycult Biotechnology, Frontstraat 2A, 5405 PB Uden, The Netherlands; 15 Department of Clinical Biochemistry and Immunology, Addenbrooke’s Hospital, Cambridge, UK; 16 Respiratory Department, King’s College Hospital NHS Foundation Trust, UK. Department of Clinical Virology, Addenbrookes, UK; 17 Department of Infectious Diseases, Cambridge University Hospitals NHS Trust, Cambridge, UK; 18 Department of Immunology, Cambridge University Hospitals NHS Trust, Cambridge, UK; 19 Cambridge University Hospitals NHS Foundation Trust, Department of Microbiology, Cambridge, UK; 20 Cambridge University Hospitals NHS Foundation Trust, Cambridge, UK; 21 Institute of Microbiology and Infection, University of Birmingham, Birmingham, UK; 22 Belfast Health and Social Care Trust, Belfast, Northern Ireland UK; 23 Regional Immunology Service, Belfast Health and Social Care Trust, Belfast, Northern Ireland UK; 24 NIHR BioResource and NIHR Cambridge Biomedical Research Centre, Cambridge Biomedical Campus, Cambridge, UK; 25 Department of Haematology, University of Cambridge School of Clinical Medicine, Cambridge Biomedical Campus, Cambridge, UK; 26 NHS Blood and Transplant, Cambridge Biomedical Campus, Cambridge, UK; 27 NHS Blood and Transplant, Oxford, UK; 28 NHS Blood and Transplant, London, UK; 29 Radcliffe Department of Medicine and BRC Haematology Theme, University of Oxford, John Radcliffe Hospital, Oxford, UK; 30 Radiology, Papworth Hospital, Cambridge, UK; 31 Cancer Research UK Cambridge Institute, Cambridge Biomedical Campus, Cambridge, UK

**Keywords:** Antivirals, SARS-CoV-2, Primary immunodeficiency disorders, Viral infection

## Abstract

The response to the coronavirus disease 2019 (COVID-19) pandemic has been hampered by lack of an effective severe acute respiratory syndrome coronavirus 2 (SARS-CoV-2) antiviral therapy. Here we report the use of remdesivir in a patient with COVID-19 and the prototypic genetic antibody deficiency X-linked agammaglobulinaemia (XLA). Despite evidence of complement activation and a robust T cell response, the patient developed persistent SARS-CoV-2 pneumonitis, without progressing to multi-organ involvement. This unusual clinical course is consistent with a contribution of antibodies to both viral clearance and progression to severe disease. In the absence of these confounders, we take an experimental medicine approach to examine the in vivo utility of remdesivir. Over two independent courses of treatment, we observe a temporally correlated clinical and virological response, leading to clinical resolution and viral clearance, with no evidence of acquired drug resistance. We therefore provide evidence for the antiviral efficacy of remdesivir in vivo, and its potential benefit in selected patients.

## Abstract (web summary)

Remdesivir is under evaluation for treatment of COVID-19 in clinical trials. Here, the authors report results of remdesivir treatment in a patient with COVID-19 and the genetic antibody deficiency XLA. They show a temporally correlated clinical and virological response, suggesting that remdesivir can reduce SARS-CoV-2 replication in patients.

## Introduction

The prodrug nucleoside analog remdesivir is a broad-spectrum antagonist of viral RNA-dependent RNA polymerase (RdRp) enzymes, leading to inhibition of SARS-CoV-2 replication in vitro^1–3^ and pre-clinical benefit in a macaque model of COVID-19.^4^ Two recent RCTs have tested the efficacy of remdesivir in patients. The first was underpowered, and failed to show clinical benefit.^5^ Preliminary data from the second showed a statistically significant reduction in illness duration, and a trend to reduced mortality.^6^ No convincing evidence of virological efficacy was reported in either study.

Although RCTs provide the gold-standard for evaluation of the efficacy of new therapeutic interventions, comparing the average responses of patients in heterogeneous treatment and control groups may mask the potential benefits for individual patients. Evaluation of therapeutics for COVID-19 is particularly complicated by the highly variable clinical course. Furthermore, as well as mediating clearance of SARS-CoV-2, the immune response may also contribute to severe COVID-19 pathology, independent of viral replication.

It is therefore unclear whether the limited response to remdesivir observed in RCTs reflects inadequate in vivo antiviral activity, or the need for concurrent immunomodulation. To minimize heterogeneity attributable to the immune response, we therefore take a reductionist, experimental medicine approach to evaluate the efficacy of remdesivir for treatment of COVID-19 in vivo, by studying a rare patient in whom the contribution of humoral (antibody-dependent) immunity to viral clearance and immunopathology is controlled genetically by the primary immunodeficiency XLA.

## Results

### Uncomplicated persistent COVID-19 pneumonitis in a patient with XLA

XLA is caused by mutations in the gene encoding Bruton’s tyrosine kinase (*BTK*), leading to an absence of mature B lymphocytes and immunoglobulins (antibodies). The subject of this study is a 31-year-old man with XLA, whose past medical history is summarized in the Methods. His illness began with fever, cough, nausea, and vomiting. On day 19, a nasopharyngeal/throat swab was positive for SARS-CoV-2 RNA, and he commenced treatment with hydroxychloroquine and azithromycin (Supplementary Fig. 1). His symptoms persisted, and a repeat nasopharyngeal/throat swab on day 28 remained positive for SARS-CoV-2 RNA.

**Fig. 1:**
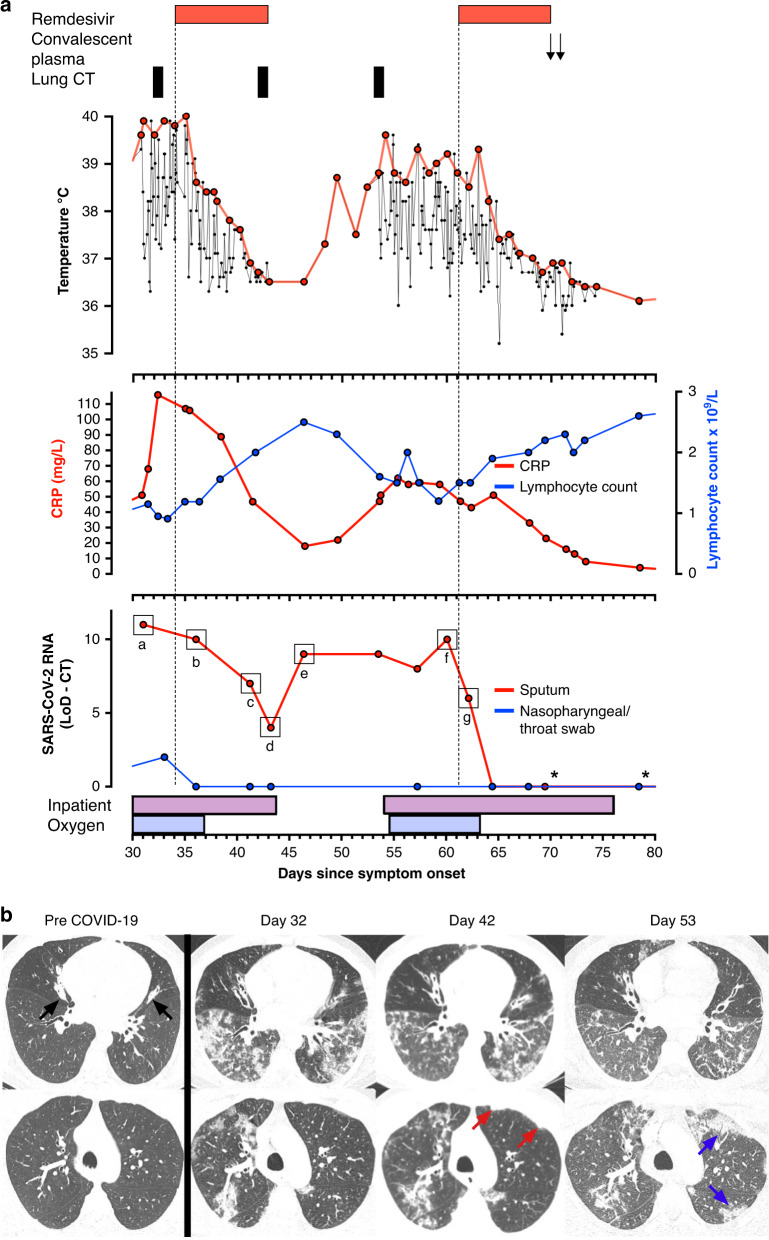
Clinical and virological assessment of the response to remdesivir. **a** Temperature, CRP, total lymphocyte count, and cycle-threshold (CT) value for viral RNA amplification from patient samples, deducted from the CT value at the limit of detection (LoD) of the assay, plotted by day since symptom onset, aligned with clinical interventions. Samples tested in the clinical laboratory at the Royal London Hospital are highlighted (*). Indicated viral isolates **a**–**g** were analyzed by Nanopore sequencing (Supplementary Fig. 2 and Table 5). *n* = 1 biologically independent samples. **b** Computerized tomography (CT) images at the level of the inferior pulmonary veins (top) and aortic arch (bottom) obtained at indicated time points. Prior to COVID-19 infection, there is moderate bronchiectasis, mucoid impaction and small airway obstruction (black arrows) in the middle lobe and lingula, but the remainder of the lungs are clear (Pre-COVID-19). Shortly before the first course of remdesivir, extensive ground-glass opacification and patchy consolidation with a lower lobe predominance is seen, together with perivascular consolidation in the right upper lobe (Day 32). Nine days after initiation of remdesivir treatment there is improvement in the lower zone ground-glass opacity and consolidation, but persistent consolidation in the right upper lobe and mild progression of the left upper lobe subpleural consolidation (red arrows) (Day 42). 10 days after completion of the first course of remdesivir treatment, following relapse of symptoms and fever, there is further improvement in the lower zone ground-glass opacification, but progressive subpleural consolidation, particularly in the left upper lobe (blue arrows) (Day 53).

The patient was admitted to hospital on day 30 because of worsening dyspnea, and supplemental oxygen was commenced (Fig. 1a). Blood tests showed a rising CRP and mild lymphopaenia, elevated IL-6, IL-10, TNF-α, and IFN-γ, but normal d-Dimer and troponin, with no evidence of significant coagulopathy, renal or liver dysfunction (Fig. 1 and Tables 1–4). Blood and sputum cultures for bacteria remained negative throughout the course of the illness, procalcitonin was repeatedly normal, and two trials of broad-spectrum intravenous antibiotics had no improved effect (Supplementary Fig. 1). A sputum sample remained positive for SARS-CoV-2 RNA, and a CT chest scan showed widespread patchy ground-glass opacity in the lower lobes, consistent with COVID-19 pneumonitis (Fig. 1b).

**Table 1:** Results from the clinical biochemistry laboratory, collated from readings at the indicated stages of the patient’s illness.

	Days after inpatient admission			
	Pre-remdesivir: day 30–31	Post first course remdesivir: day 45–51	Second course remdesivir: day 60	Post second course remdesivir: day 67
Serum sodium (mmol/litre)	135 (133–146)	139 (133–146)	134 (133–146)	
Serum potassium (mmol/litre)	3.6 (3.5–5.3)	4.3 (3.5–5.3)	3.4 (3.5–5.3)	
Serum urea (mmol/litre)	3.8 (2.5–7.8)	2.7 (2.5–7.8)	2.1 (2.5–7.8)	
Serum creatinine (µmol/litre)	75 (62–115)	77 (62–115)	**56** (62–115)	
Albumin (g/litre)	**28** (36–50)			
Total bilirubin (µmol/litre)	9 (0–20)		5 (0–20)	
Alkaline phosphatase (U/litre)	66 (30–130)		77 (30–130)	
Alanine transaminase (U/litre)	26 (10–49)		13 (10–49)	
Triglyceride (mmol/litre)	**3.17** (0.3–1.8)			

Abnormal results are shown in bold. Numbers in parentheses indicate reference values of the corresponding measurements.

**Table 2:** Results from the clinical hematology laboratory, collated from readings at the indicated stages of the patient’s illness.

	Days after inpatient admission			
	Pre-remdesivir: day 30–31	Post first course remdesivir: day 45–51	Second course remdesivir: day 60	Post second course remdesivir: day 67
d-Dimer (ng/ml) d-Dimer (ml/L FEU)	201 (0–230)	**0.66** (0–0.5)	**0.77** (0–0.5)	
Fibrinogen (g/litre)	**6.33** (1.46–3.33)	**7.3** (1.46–3.33)	**6.61** (1.46–3.33)	
Ferritin (µg/L)	**1063.5** (22–322)	**410** (22–322)	**546** (22–322)	
Lactate dehydrogenase (U/litre)	**620** (120–246)			
NT pro BNP (pg/ml)	12			
High sensitivity troponin (ng/litre)	5.8 (0–58.1)			
APTT (sec)	36.1 (28.2–36.6)		28 (28.2–36.6)	
PT (sec)	**15.6** (10.8–13.3)	11.2 (10.8–13.3)	12 (10.8–13.3)	

Abnormal results are shown in bold. Numbers in parentheses indicate reference values of the corresponding measurements. *APTT* activated partial thromboplastin time, *PT* prothrombin time.

**Table 3:** Results from the clinical immunology laboratory, collated from readings at the indicated stages of the patient’s illness.

	Days after inpatient admission			
	Pre-remdesivir: day 30–31	Post first course remdesivir: day 45–51	Second course remdesivir: day 60	Post second course remdesivir: day 67
IgG (g/litre)	9.6 (6.34–18.11)	13.8 (6.34–18.11)		16.5 (6.34–18.11)
IgA (g/litre)	**<0.3** (0.8–2.8)	**<0.05** (0.8–2.8)		**<0.05** (0.8–2.8)
IgM (g/litre)	**<0.2** (0.5–1.9)	**<0.05** (0.5–1.9)		**<0.05** (0.5–1.9)
Complement C3 (g/litre)	**2.26** (0.75–1.65)			
Complement C4 (g/litre)	**0.66** (0.14–0.54)			
Alternative pathway AP100 (%)	>129 (66–129)			
Classical pathway CH100 (U/ml)	>911 (392–1019)			
TNF Alpha (pg/ml)	**15.65** (0–5)			
IL-1 beta (pg/ml)	0.76 (0–3.1)			
IL-10 (pg/ml)	**2.91** (0–1)			
IFN- gamma (pg/ml)	**19.19** (< 10)			
IL-6 (pg/ml)	**31.8** (0–2)			
*CD3*^*+*^ *T cells*				
%	91		93	
Total (×10^9^ /litre)	1.21 (0.7–2.1)		1.61 (0.7–2.1)	
*CD4*^*+*^ *T cells*				
%	62		58	
Total (×10^9^ /litre)	0.82 (0.2–1.4)		1.01	
*CD8*^*+*^ *T cells*				
%	29		34	
Total (×10^9^ /litre)	0.39 (0.2–0.9)		0.58	
*CD19*^*+*^ *B cells*				
%	0		0	
Total (×10^9^ /litre)	0 (0.1–0.5)		0	
*CD56*^*+*^ *NK cells*				
%	9		6	
Total (×10^9^ /litre)	0.12 (0.09–0.6)		0.11	

Abnormal results are shown in bold. Numbers in parentheses indicate reference values of the corresponding measurements.

**Table 4:** Results from the clinical microbiology laboratory, collated from readings at the indicated stages of the patient’s illness.

	Days after inpatient admission			
	Pre-remdesivir: day 30–31	Post first course remdesivir: day 45–51	Second course remdesivir: day 60	Post second course remdesivir: day 67
Procalcitonin (ng/ml)	0.15 (0–0.5)	0.06 (0–0.5)		
HIV RNA	Not detected			
Adenovirus DNA	Not detected			
Human metapneumovirus RNA	Not detected			
Influenza A generic	Not detected			
Influenza B RNA	Not detected			
Parainfluenza virus RNA	Not detected			
RSV RNA	Not detected			
Picornavirus RNA	Not detected			

Numbers in parentheses indicate reference values of the corresponding measurements.

Immunocompetent adults with severe COVID-19 typically exhibit a monophasic acute illness, with hospital admission and progressive respiratory failure 7–10 days after symptom onset^7,8^. In contrast, our patient exhibited a very unusual pattern of SARS-CoV-2 infection, with persistent fever and pneumonitis for >30 days, but without progression to acute respiratory distress syndrome (ARDS) or multi-organ involvement. This relatively stable baseline allowed the detailed assessment of clinical, virological and immune responses during two independent challenges with remdesivir.

### Clinical response to remdesivir

On day 34, hydroxychloroquine and azithromycin were discontinued, and the patient commenced a 10 day course of remdesivir. His fever and dyspnea improved within 36 hours of the first dose, nausea and vomiting ceased, and rising oxygen saturation allowed discontinuation of supplemental oxygen. This dramatic clinical response was accompanied by a progressive decrease in CRP, a rise in total lymphocyte count, and an improvement in ground-glass opacification on repeat CT chest (Fig. 1a, b). The patient was therefore discharged on day 43.

Seven days after discharge, his fever, dyspnea, and nausea returned. He was readmitted to hospital on day 54, and supplemental oxygen was commenced. A further sputum sample was positive for SARS-CoV-2 RNA, and a CT pulmonary angiogram showed evidence of ongoing pneumonitis (Fig. 1a, b). Consistent with a recrudescence of COVID-19, the patient’s CRP increased, and his lymphocyte count fell. On day 61, he therefore began treatment with a further 10 day course of remdesivir. Once again, his symptoms rapidly improved, his fever and requirement for supplemental oxygen resolved, and his CRP and lymphocyte count normalized (Fig. 1a).

The patient therefore exhibited a marked clinical response, tightly correlated with the administration of remdesivir, over two independent challenges. Because of his underlying immunodeficiency, protracted illness, and relapse after the first course of remdesivir, he was further treated with two units of convalescent plasma on days 69 and 70, to provide secondary prophylaxis against SARS-CoV-2 infection (Fig. 1a). He was discharged 3 days later, and has remained apyrexial and asymptomatic over a further 28 days of follow-up.

### Virological response to remdesivir

SARS-CoV-2 RNA was identified on nasopharyngeal/throat swabs early in the patient’s illness, but became undetectable on these samples from day 36 (Fig. 1a). Because of his underlying bronchiectasis, the patient habitually expectorates small volumes of sputum. SARS-CoV-2 RNA was readily detectable in these samples until day 64, 4 days into his second remdesivir course, allowing non-invasive monitoring of his lower respiratory tract. Samples from blood, urine, feces, and a rectal swab were all negative.

Strikingly, levels of SARS-CoV-2 RNA in sputum fell progressively during the patient’s first course of remdesivir, corresponding with his clinical response (Fig. 1a). Nonetheless, SARS-CoV-2 RNA remained detectable at low levels. Upon cessation of remdesivir treatment, levels of SARS-CoV-2 RNA increased again in parallel with the recrudescence of symptoms. The effect of the second course of remdesivir was even more rapid and complete, with SARS-CoV-2 RNA becoming undetectable after 4 days (Fig. 1a). The patient therefore exhibited a dramatic virological response, tightly correlated with both the administration of remdesivir and resolution of his symptoms.

To confirm that the recurrent detection of SARS-CoV-2 RNA reflected viral persistence, rather than reinfection, isolates were sequenced regularly over the course of the patient’s illness (Fig. 1a and Supplementary Fig. 2 and Table 5). All isolates belonged to the B2.6 lineage, a very uncommon lineage globally with only 47 isolates in total from the UK, making reinfection extremely unlikely. Compared with the Wuhan-Hu-1 reference sequence, 17 single-nucleotide polymorphisms (SNPs) were present in at least one isolate (Supplementary Fig. 2). Among these, the only SNP in the remdesivir target gene *nsp12*, encoding the RdRp, was a synonymous variant (C14805T), present at equivalent abundance across all isolates. Despite the combination of underlying immunodeficiency, prolonged viral shedding and repetitive treatment, there was therefore no evidence of acquired remdesivir resistance.

**Table 5:** Viral sequence accession numbers that were compared to the reference GenBank accession MN908947.3.

Virus name	COG-UK ID	GISAID accession	ENA accession	ENA hyperlink
hCoV-19/England/CAMB-7FE20/2020	CAMB-7FE20	EPI_ISL_444421	SAMEA6958574	https://www.ebi.ac.uk/ena/browser/view/SAMEA6958574
hCoV-19/England/CAMB-82C3F /2020	CAMB-82C3F	EPI_ISL_438672	SAMEA6957692	https://www.ebi.ac.uk/ena/browser/view/SAMEA6957692
hCoV-19/England/CAMB-1AC102/2020	CAMB-1AC102	EPI_ISL_444373	SAMEA6960187	https://www.ebi.ac.uk/ena/browser/view/SAMEA6960187
hCoV-19/England/CAMB-1AD7F0/2020	CAMB-1AD7F0	EPI_ISL_448012	SAMEA6961791	https://www.ebi.ac.uk/ena/browser/view/SAMEA6961791
hCoV-19/England/CAMB-1B2093/2020	CAMB-1B2093	EPI_ISL_453003	SAMEA6964413	https://www.ebi.ac.uk/ena/browser/view/SAMEA6964413
hCoV-19/England/CAMB-1B2CF9/2020	CAMB-1B2CF9	EPI_ISL_456720	SAMEA6965179	https://www.ebi.ac.uk/ena/browser/view/SAMEA6965179
hCoV-19/England/CAMB-1AC366/2020	CAMB-1AC366	EPI_ISL_584283	SAMEA7459180	https://www.ebi.ac.uk/ena/browser/view/SAMEA7459180

These data are available from https://www.cogconsortium.uk/data/.

### Contribution of antibodies to clearance of SARS-CoV-2

XLA is the prototypic genetic disorder of the humoral immune system, and the most obvious explanation for our patient’s failure to clear his infection spontaneously is his lack of antibodies to SARS-CoV-2. Patients with XLA are known to be at risk from persistent RNA viral infections, particularly chronic enteroviral meningoencephalitis^9^. This risk is mitigated by immunoglobulin replacement therapy, including antibodies to enteroviruses.^10^ Conversely, available pooled immunoglobulin preparations antedate the COVID-19 pandemic, and lack specific SARS-CoV-2 antibodies.

**Fig. 2:**
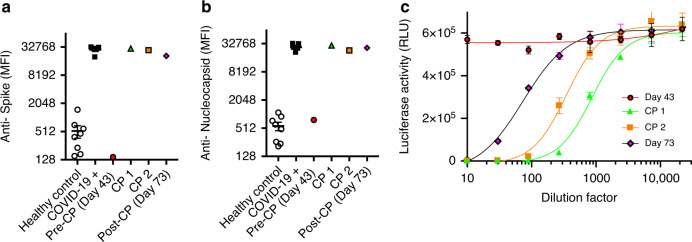
Kinetic assessment of the antigen-specific humoral responses. Serological reactivity to SARS-CoV-2 spike **a** and nucleocapsid **b** antigens of patient sera obtained at indicated time points, the first (CP 1) and second (CP 2) infusions of convalescent plasma (CP), sera from healthy controls (*n* = 8 biologically independent subjects) and patients (*n* = 5 biologically independent subjects) with PCR-confirmed COVID-19 (MFI, mean fluorescence intensity; Mean and SEM). **c** Neutralisation activity against SARS-CoV-2 spike-pseudotyped lentiviral particles of patient sera obtained at indicated time points, compared with the first (CP 1) and second (CP 2) infusions of convalescent plasma (CP). (*n* = 1 independent biological samples, *n* = 3 technical replicates from each sample; mean and SD). Data from one independent experiment.

We therefore assessed the humoral immune response to SARS-CoV-2 in our patient. As expected, despite maintenance of regular immunoglobulin replacement, antibodies to SARS-CoV-2 spike and nucleocapsid proteins were undetectable by immunoassay prior to treatment with convalescent plasma (Fig. 2a, b), and no neutralization activity was observed against lentiviral particles pseudotyped with SARS-CoV-2 spike protein (Fig. 2c). Following administration of convalescent plasma, antibody levels and neutralisation activity rose commensurately (Fig. 2a–c).

### CD8+ T-cell response to SARS-CoV-2

Together with CD8+ T cells, antibodies are known to contribute to the control of other RNA viruses, including HIV^11^, Hepatitis C^12^, and LCMV infection of mice^13,14^. Some recent reports of patients with XLA and COVID-19^15–17^ did not describe persistent clinical disease. This suggests that, similar to the LCMV model, CD8+ T-cell immunity can sometimes compensate for humoral deficiency in the control SARS-CoV-2. We therefore assessed the antigen-specific CD8+ T- response to SARS-CoV-2 in our patient.

**Fig. 3:**
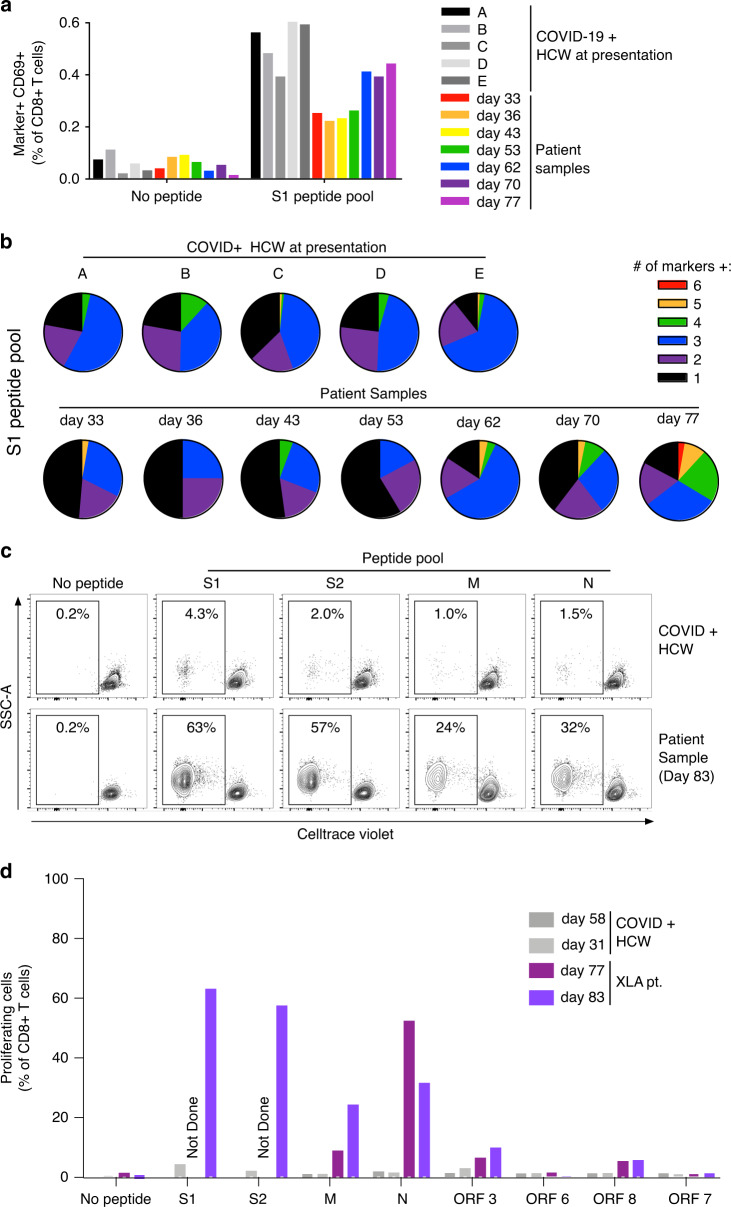
Kinetic assessment of the antigen-specific CD8+ T-cell responses. **a** % CD8+ T cells expressing activation makers after incubation ± a peptide pool covering the SARS-CoV-2 S1 protein. Patient samples obtained at indicated time points are compared with HCWs with PCR-confirmed COVID-19 at presentation (*n* = 5 biologically independent subjects). **b** Number of activation markers expressed by S1-responsive CD8+ T cells from **a**. Representative flow cytometry dot plots **c** and % proliferating CD8+ T cells **d** after stimulation ± peptide pools covering the indicated SARS-CoV-2 proteins. Patient samples are compared with HCWs (*n* = 2 biologically independent subjects) with PCR-confirmed COVID-19 at indicated time points. Data from one independent experiment.

Immunodominant CD8+ T-cell epitopes of SARS-CoV-2^18^ and other coronaviruses^19,20^ are contained within the spike protein. We first measured the quality of the CD8+ T-cell response by flow cytometry for antigen-stimulated effector protein expression^21^ (Fig. 3a). The frequency of spike-specific CD8+ T cells within the circulation was comparable to age-matched healthcare workers with acute COVID-19, trending upwards over the course of infection. Increasing polyfunctional effector capacity was evident from day 62 (Fig. 3b), particularly the appearance of TNF-α and IFN-γ producing CD8+ T cells (Supplementary Fig. 3).

We further assessed the coverage of the patient’s antigen-specific response by measuring CD8+ T-cell proliferation to peptide pools covering the S1, S2, M, N, ORF3, ORF6, ORF8, and ORF7 SARS-CoV-2 proteins, following viral clearance. At days 77 and 83, the patient had striking responses to all viral peptides relative to age-matched COVID-19 infected HCWs (Fig. 3c, d), likely reflecting the prolonged duration of antigen exposure. Taken together, these data confirm the presence of a robust CD8+ T-cell response to SARS-CoV-2. Although insufficient to resolve the infection spontaneously, this likely contributed to the clearance of virus during the second course of remdesivir.

### Role of antibodies in immunopathogenesis of COVID-19

As well as their role in the antiviral response, antibodies have the potential to cause immune pathology^22^. In a retrospective study of patients with SARS-CoV, the development of neutralizing antibodies early in infection correlated with worse disease outcome^23^, and high antibody titers are associated with severe disease in COVID-19^24^. Our patient’s lack of SARS-CoV-2 antibodies may therefore explain why, despite his persistent infection, he did not progress to ARDS or multi-organ involvement.

Antigen–antibody complexes are able to activate the classical complement pathway, with release of the anaphylatoxins C3a and C5a and formation of the multi-subunit terminal complement complex (TCC). C3-deficiency reduces lung pathology in a mouse model of SARS-CoV^25^, complement activation is a feature of COVID-19^26^, and deposition of TCC is observed in the microvasculature of patients with severe disease^27^.

**Fig. 4:**
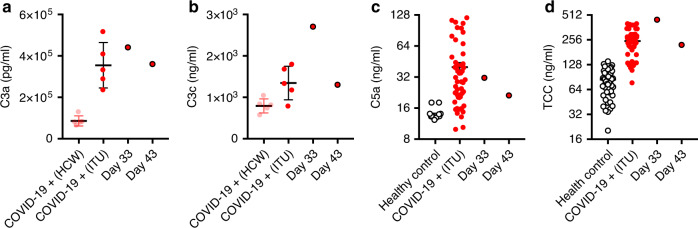
Assessment of complement activation. Concentrations of complement cleavage products C3a **a**, C3c **b**, and C5a **c**, and circulating terminal complement complex (TCC) **d** at indicated time points, compared with healthy controls or patients with PCR-confirmed COVID-19 (healthcare workers, HCW or patients with severe disease admitted to the intensive therapy unit, ITU; mean and SEM are shown). For **a** and **b** HCW, *n* = 5 biologically independent subjects and ITU *n* = 5 biologically independent subjects. For **c** Healthy controls, *n* = 12 biologically independent subjects and ITU *n* = 51 biologically independent subjects. **d** Healthy controls, *n* = 67 biologically independent subjects and ITU *n* = 50 biologically independent subjects. Data from one independent experiment.

We therefore assessed the level of complement activation in our patient. Despite the lack of disease progression, levels of C3a C3c, C5a, and TCC were markedly elevated, comparable to levels seen in COVID-19 patients admitted to the intensive care unit (Fig. 4a–d). Although complement activation correlates with and may be required for the development of ARDS and/or multi-organ failure, complement activation alone is therefore insufficient for disease progression, and the classical pathway is not required for complement activation in COVID-19.

## Discussion

Taken together, our observations confirm that remdesivir is a potent antiviral agent for treating SARS-CoV-2 infection in vivo. It is striking that previous reports from immunocompetent patients have generally not demonstrated the profound clinical and virological responses observed in this study. This is likely because, among the hospitalized patients included in RCTs^5,6^ or compassionate use programs^28^ to date, progression or resolution of disease has been determined not by the level of SARS-CoV-2 replication, but by the evolution of immune pathology. Although remdesivir was given chronologically late in our patient’s disease, he had not developed ARDS or multi-organ involvement. The utility of remdesivir in immunocompetent patients may therefore be maximized by early treatment in the most at risk, or by combination with targeted immunomodulatory therapies in the most sick.

Other reports of patients with XLA and COVID-19 have described mild disease, of heterogenous duration.^15–17,29,30^ Similar to recent cases in which convalescent plasma was used for treatment^29,30^, the persistent disease observed here strongly suggests that antibodies contribute to the control of SARS-CoV-2, at least in some patients. Alongside the failure of specific antibody production, *BTK* is also expressed in other immune cells^31,32^, and monocyte targeting by *BTK* inhibition can ameliorate COVID-19^33^. Nonetheless, monocyte dysfunction in patients with XLA is corrected by adequate replacement immunoglobulin^34^, and this treatment effectively mitigates the immunodeficiency seen in routine clinical practice.

The lack of disease progression observed in this patient suggests that antibodies may also contribute to immune pathology in COVID-19, and administration of convalescent plasma has the potential to trigger an inflammatory response^30^. Aside from activating complement, antibodies may interact with Fc receptors on immune cells to drive macrophage activation and inflammatory cytokine production^35^. Strategies that target these interactions include plasmapheresis, or blockade of Fc receptors by saturating doses of intravenous immunoglobulin. The potential for both beneficial and deleterious effects of SARS-CoV-2 antibodies revealed by our study suggests that these interventions, like the administration of convalescent plasma, should be tailored to individuals or subgroups of patients with distinct clinical characteristics.

## Methods

### Oversight

The study was approved by the East of England—Cambridge South national institutional ethics review board (17/EE/0025). The patient provided written informed consent. Additional healthy controls, patients and healthcare workers with COVID-19 provided written informed consent and were enrolled to the NIHR BioResource Centre Cambridge (17/EE/0025) and the Oxford Gastrointestinal Illness Biobank (16/YH/0247).

### Patient characteristics

The subject of this study is a 31-year old man, born in the UK to parents of Pakistani origin, and diagnosed with XLA at the age of 12. He has a family history of XLA affecting his brother, four maternal uncles and a male maternal cousin. Following investigation for recurrent chest infections, he was found to lack mature B cells and circulating immunoglobulins, and confirmed to have inherited the familial c. 1430delT mutation in the gene encoding *BTK*. Because of prior incomplete adherence to replacement immunoglobulin therapy, he developed middle lobe bronchiectasis.

### Remdesivir treatment

The first course of remdesivir was administered as part of a Gilead SIMPLE study (NCT04292899), the second course was provided as part of the Gilead Expanded Access Program (NCT04323761). In each case, the patient received an initial dose of 200 mg IV, followed by niine daily doses of 100 mg IV (10 days total).

### Convalescent plasma

Convalescent plasma was collected from individuals with previous, laboratory-confirmed SARS-CoV-2 infection at least 28 days after resolution of symptoms using established infrastructure and standard UK donor selection guidelines, as previously described^36^. Signed consent was obtained from each donor at the time of donation using NHS Blood and Transplant-approved consent forms, in accordance with Blood Safety and Quality Regulations enforced by the Medicines & Healthcare products Regulatory Agency.

In brief, a total of at least 540 ml of plasma (containing < 1 × 10^6^ leukocytes per component) was collected via plasmapheresis from each donor, divided into two units, rapidly frozen and stored at −25 °C. Donor blood samples were tested for SARS-CoV-2 RNA (Public Health England) and antibodies (EUROIMMUN (IgG) assay, PerkinElmer, London, UK). A signal to cutoff (S/CO) ratio of 9.1 in the EUROIMMUN assay was previously shown to identify donations with a neutralizing antibody titre of ≥1:100 in a SARS-CoV-2 (isolate England/2020) microneutralisation assay with a specificity of 100%.^36^ Both donations used in this study were collected in May 2020 and confirmed to be negative for SARS-CoV-2 RNA. EUROIMMUN S/CO ratios were 37.171 (first unit, 290 ml, administered on day 69) and 7.271 (second unit, 283 ml, administered on day 70), respectively. For the second unit (lower S/CO ratio), the neutralizing antibody titre was confirmed to be ≥1:100 using the SARS-CoV-2 microneutralisation assay.^36^ Plasma was defrosted using a 37°C waterbath, and transfused within 4 hours of defrosting.

### Patient sampling

Upper respiratory tract samples (nasopharyngeal/throat swabs) were collected in viral transport medium according to Public Health England (PHE) guidelines. Lower respiratory samples (sputum) were collected in universal containers and extracted following mucolysis with Mucolyse PL.701 sputum liquefying agent (Pro-Lab Diagnostics). To minimize RNA degradation, the time between sample collection and processing did not exceed 2 hr.

Peripheral blood mononuclear cells (PBMCs) were isolated using Leucosep tubes (Greiner Bio-One) with Histopaque-1077 (Sigma) by centrifugation at 800 × *g* for 15 mins at room temperature. PBMCs at the interface were collected and rinsed twice with autoMACS running buffer (Miltenyi Biotech). All samples were processed within 4 h of venepuncture. Samples were then resuspended in freezing medium (FBS 90% supplemented with dimethyl sulfoxide (DMSO) 10%), frozen overnight at −80 °C in a Mr Frosty cell freezing containger (Nalgene) then transferred to liquid nitrogen for long term storage.

### SARS-CoV-2 molecular testing

Sample testing was carried out using an in-house real time uniplex RT-PCR diagnostic assay for the detection of SARS-CoV-2 in the PHE Clinical Microbiology and Public Health Laboratory at Addenbrooke’s Hospital, Cambridge. This assay targets a 222 base-pair region of the SARS-CoV-2 *nsp12* gene (encoding RdRp), and has been validated for clinical use.

In brief, nucleic acid extraction was undertaken using the NUCLISENS easyMAG platform (Biomerieux, Marcy L-Etoile), in accordance with the manufacturer’s instructions. Nucleic acids were extracted from 500 μL sample, with a dilution of MS2 bacteriophage (4600pfu per extraction) added pre-extraction to act as an internal extraction and inhibition control. Molecular grade water was used as a negative control. Positive control material, BetaCoV/England/02/2020, was obtained from PHE Colindale (essentially, purified viral RNA diluted to give a cycle-threshold (CT) value of 26–28).

The RdRp gene was detected using primers ATGGGTTGGGATTATCCTAAATGTGA and AGCAGTTGTGGCATCTCCTGATGAG with a FAM-labeled MGB RdRp Probe ATGCTTAGAATTATGGCCTCAC. The internal extraction control was detected using the MS2 forward primer TGGCACTACCCCTCTCCGTATTCACG, the MS2 reverse primer GTACGGGCGACCCCACGATGAC and a ROX-BHQ2 labeled MS2 probe CACATCGATAGATCAAGGTGCCTACAAGC. Amplification reactions and detection of PCR products were performed using the RotorgeneTM PCR instrument. A typical reaction contained 400 nM of forward and reverse primers for the RdRp gene and 200 nM of the MS2 internal control forward and reverse primer pair, along with 120 nM of the RdRp and MS2 probes. The cycle conditions were as follows: 25 °C 2 mins, 50 °C 15 mins, 95 °C 2 mins followed by 45 cycles of 95 °C and 60 °C. Samples that generated a CT value ≤36, defined as 0.01 fluorescence units as per the RotorgeneTM manufacturer’s instructions, were considered positive (roughly equivalent to eight genome copies, the lower limit of detection for the assay). Where indicated, additional samples were tested in the clinical laboratory at the Royal London Hospital, London.

### Viral sequencing and bioinformatics

Samples were analyzed by Nanopore sequencing as part of the COG-UK sequencing project, following the ARTICnetwork V3 protocol (10.17504/protocols.io.bbmuik6w), and assembled using the ARTICnetwork assembly pipeline (https://artic.network/ncov-2019/ncov2019-bioinformatics-sop.html). The accession numbers of the sequences included in this study are available in Table 5. Median genome depth of coverage was 1346×. Consensus FASTA sequences were analyzed after QC filtering, de-duplication and matching with metadata. Variants were initially assessed using the ARTICnetwork assembly pipeline VCF output files, with a SNP being called when >50.1% frequency at a single base. These were then independently confirmed using iVar analysis (https://andersen-lab.github.io/ivar/html), set to a quality threshold of Q20 and a minimum frequency of 10%, with a SNP being called when >50.1% of the reads at a particular position differed from the reference sequence, Wuhan-Hu-1 (GenBank Accession MN908947.3). The location of each SNP was examined to identify mutations in the *nsp12* gene, encoding RdRp (the target of remdesivir treatment). For the kinetic assessment, proportions of each variant as a fraction of all reads at their corresponding locations (minimum depth 20×) were plotted as a proportional stacked area chart using ggplot2 and RStudio.

### Intracellular cytokine staining

Cryopreserved PBMCs were rapidly thawed, washed in R10 media (RMPI-1640+10% FBS+1% Pen/Strep), and 10^6^ cells added to wells of a 96-well U-bottom plate. Cells were stimulated with 2g/ml overlapping S1 or M peptide pools, or left unstimulated, for 2 h at 37 °C, 5% CO2. Anti-CD107a-BV785 (1:100 dilution, clone H4A3), anti-CD28 (clone CD28.2, 1 µg/ml) and anti-CD49d (clone R1-2, 1 µg/ml) to all wells at the time of peptide addition. After 2 h, brefeldin A (5 µg/ml) and monensin (2 µM) were added, and cells were incubated at 37 °C, 5% CO_2_ for an additional 16 hr. After stimulation, cells were washed two times with fluorescence-activated cell sorting (FACS) buffer (PBS+1 mM EDTA+ 0.05% BSA). Surface staining was performed at 4 °C for 30 mins. Cells were then washed two times in FACS buffer, and fixed and permeabilized at 4 °C for 30 mins using BD Cytofix/Cytoperm solution. Cells were then washed twice with 1× BD Perm/Wash buffer, and stained for intracellular markers at 4 °C for 30 mins. Two further washes with 1× BD Perm/Wash buffer were performed and cells were stored in FACS buffer at 4 °C. Samples were acquired on a custom Cytek Aurora spectral analyzer (four laser; UV, violet, blue, and red) using SpectroFlo v2.2. Data were analyzed using FlowJo v. 10.6.2 and Prism v. 8.3.0.

### T-cell proliferation assay

Freshly isolated PBMCs were labeled using CellTrace Violet (Invitrogen) and stimulated with peptide pools spanning the entire S, M, N, and ORFs 3, 6, 7, and 8 SARS-CoV-2 proteins at 1 µg/ml of each overlapping peptide. Stimulation was done in Roswell Park Memorial Institute (RPMI) media (Sigma) supplemented with 10% AB serum (Sigma), 1% Pen/strep and 1% l glutamine for 7 days at 37 °C. 0.1% DMSO, representative of DMSO content in peptide stimulated wells, was used as negative control and 2 µg/ml of PHA (Sigma) served as positive control. On day 4, 100 µl of media was exchanged with 100 µl of fresh media. On day 7, cells were washed using FACS wash buffer (Biolegend) and stained with L/D Near Infra red (Invitrogen), CD3 FITC (Biolegend) at 1:50 dilution, CD4 AF700 (BD Bioscience) at 1: 100 dilution and CD8 PECY7 (Biolegend) at 1:200 dilution. Samples were subsequently fixed and acquired on a BD LSR II. Data were analyzed using flowjo v10.6.2.

### Serological assessment

*Serological reactivity to SARS-CoV-2 spike and nucleocapsid proteins*: recombinant SARS-CoV-2 nucleocapsid and spike proteins were covalently coupled to distinctive carboxylated bead sets (Luminex; Netherlands) to form a multiplex assay. For protein coupling, beads were first activated with 1-ethyl-3-[3-dimethylaminopropyl]carbodiimide hydrochloride (Thermo Fisher Scientific) in the presence of N-hydroxysuccinimide (Thermo Fisher Scientific), according to the manufacturer’s instructions, to form amine-reactive intermediates. The activated bead sets were incubated with the corresponding proteins at a concentration of 50 μg/ml in the reaction mixture for 3 h at room temperature on a rotator. Beads were washed and stored in a blocking buffer (10 mM PBS, 1% BSA, 0.05% NaN3).

Coupled bead sets were incubated with patient or control sera at a dilution of 1/100 for 1 h in 96-well filter plates (MultiScreenHTS; Millipore) at room temperature in the dark on a horizontal shaker. Fluids were aspirated with a vacuum manifold and beads were washed three times with 10 mM PBS/0.05% Tween-20. Beads were incubated for 30 mins with a PE-labeled anti–human IgG-Fc antibody (Leinco/Biotrend), washed as described above, and resuspended in 100 μl PBS/Tween-20. They were then analyzed on a Luminex analyzer (Luminex/R&D Systems) using Exponent Software V31. Specific binding was reported as mean fluorescence intensities. Stored sera collected in the diagnostic immunology laboratory prior to November 2019 were used as healthy controls. Sera collected from patients with PCR-confirmed COVID-19 were used as positive controls.

### Neutralisation activity

*Cell lines*: HEK293T (Lehner laboratory stocks) and HEK293T + ACE2 cells were cultured in Iscove’s Modified Dulbecco’s Media (Sigma) supplemented with 1% GlutaMAX™, 10% FCS, 100units/ml penicillin and 100 µg/ml streptomycin (all Thermo Fisher), at 37 °C in 5% CO_2_. HEK293T cells constitutively expressing angiotensin-converting enzyme 2 (ACE2) (HEK293T + ACE2 cells) were generated by transduction of wildtype HEK293T cells with a pHRSIN-ACE2-hygro^R^ lentivirus and selected 48 h post transduction using 100 µg/mL hygromycin B (Sigma). All cells were confirmed to be mycoplasma negative (MycoAlert, Lonza).

*Plasmids*: plasmid pCG1-SARS-CoV-2 Δ19 expressing humanized SARS-CoV-2 Δ19 spike was created by amplification of truncated spike from pCG1-SARS-2-S (a kind gift from M. Hoffmann, Infection Biology Unit, Leibniz Institute for Primate Research, Gottingen, Germany) using Phusion polymerase (NEB) and primer pair CoV2optFor (ttgtatcggatccaccatgttcgtgtttctggtgctgctg) and CoV2optD19rev (atcccgatctagatcagcagcagctgccacagctaca). The amplified product, lacking the C-terminal 19 amino acids, was digested and re-cloned into the pCG1 vector using BamHI-XbaI sites. pHRSIN-ACE2-hygro^R^ was produced by cloning into KpnI-XhoI-digested pHRSIN-pSFFV MCS(+) pGK-Hygro using NEBuilder HiFi DNA assembly (NEB) of the ACE2 gene amplified from HepG2 mRNA, using Phusion polymerase (NEB) and primer pair ACE2-cDNA_Fwd (cgcccgggggggatccactaggtaccatgtcaagctcttcctggctcc) and ACE2_cDNA_Rev (ctagagtcgcggccgctctactcgagctaaaaggaggtctgaacatcatcagtgttttg), following reverse transcription using Superscript III (Thermo Fisher) and oligo (dT)_15_ (Promega). pHRSIN-firefly luc-Puro^R^ was produced by ligation of the BamHI–NotI-digested firefly luciferase cDNA into BamHI-NotI-digested pHRSIN-pSFFV-EmGFP pGK puro.

*Virus production*: pseudotyped lentiviral stocks were generated through the co-transfection of HEK293T cells with a lentiviral expression vector plus the packaging plasmid pCMVΔR8.91 and either pMD.G (VSV-G) or pCG1-SARS-CoV-2 Δ19 spike plasmid, using TransIT-293 transfection reagent (Mirus) according to the manufacturer’s recommendations. Viral supernatants were harvested 48 h post transfection and cell debris was removed with a 0.45-um filter prior to use or freezing.

*Neutralisation assay*: pseudotyed pHRSIN-firefly luc-Puro^R^ lentivirus supernatants were pre-incubated with diluted serum or plasma samples at 37 °C for 60 mins, before addition of 1.25 × 10^4^ HEK293T cells to each well. Transduction efficiencies were quantified 48 h post transduction by measuring firefly luciferase activity in cell lysates using Bright-Glo™ luciferase assay system (Promega) and read in a CLARIOstar luminometer. Data were plotted using GraphPad Prism 8 (non-linear regression, (Inhibitor) vs. response–Variable slope, four parameters) and virus neutralizing antibody titre was obtained by determining the dilution factor which gave a 50% reduction in firefly luciferase levels, compared with the no serum control wells.

### Complement assays

The complement components C3a, C3c, and C5a were measured in plasma samples from patients with COVID-19 using solid-phase enzyme-linked immunosorbent assay (ELISA, Hycult Biotech) according to the manufacturer’s instructions. TCC was measured using an in-house ELISA with the anti-TCC neo-epitope antibody, ae11, used at 5 µg/ml as capture.

### Reporting summary

Further information on research design is available in the Nature Research Reporting Summary linked to this article.

## Supplementary information

Supplementary Information

Reporting Summary

## Data Availability

All relevant data are available from the authors. Viral sequence data are available from COVID-19 Genomics UK and the GSAID. The accession numbers are detailed in Table 5 and are accessible from https://www.cogconsortium.uk/data/. Source data are provided with this paper.

## Source data

Source Data
