# Supplementary material for: Treatment of COVID-19 with remdesivir in the absence of humoral immunity: a case report
Source: Nat Commun. 2020 Dec 14;11:6385. doi: 10.1038/s41467-020-19761-2 (PMC7736571; doi:10.1038/s41467-020-19761-2)
Supplement: Supplementary file 1 — Supplementary Information [file 41467_2020_19761_MOESM1_ESM.pdf]

Supplementary Figure 1. Clinical assessment of the response to remdesivir.

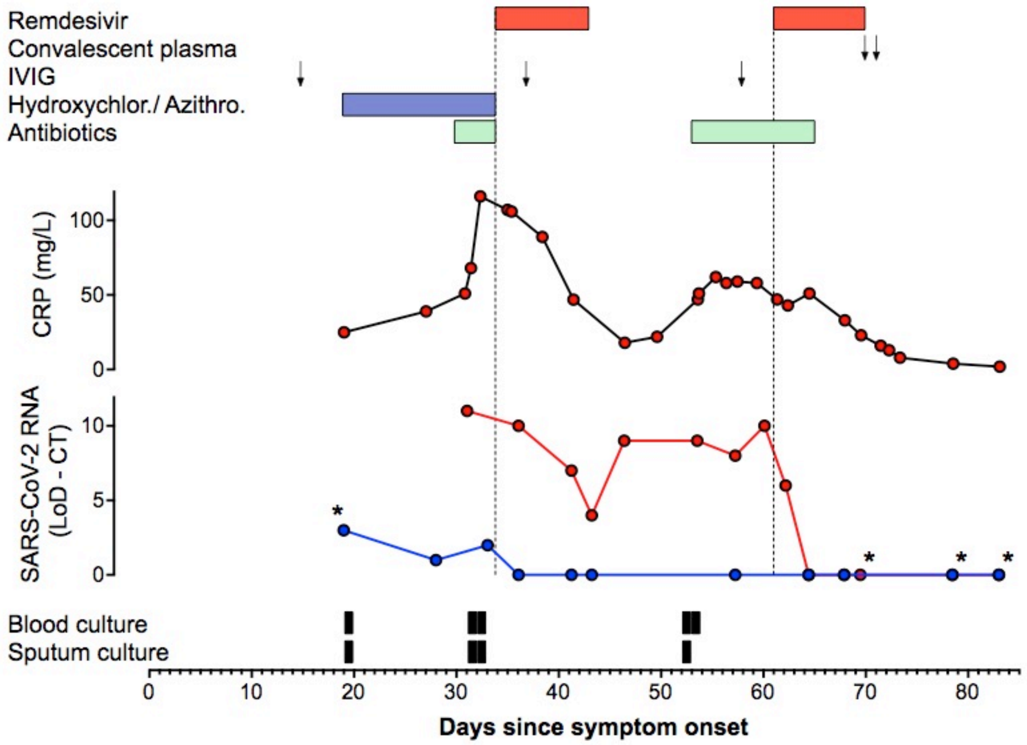

Supplementary Figure 1. As for Figure 1a (CRP) and (SARS-CoV-2 RNA) but spanning course of entire illness and indicating additional, incidental clinical interventions (IVIG, hydroxychloroquine/azithromycin, antibiotics) and days on which negative bacterial blood and sputum cultures were obtained. Antibiotics, IV meropenem (first course) or ceftriaxone and clarithromycin (second course).

Supplementary Figure 2. Identification of viral polymorphisms in sputum isolates.

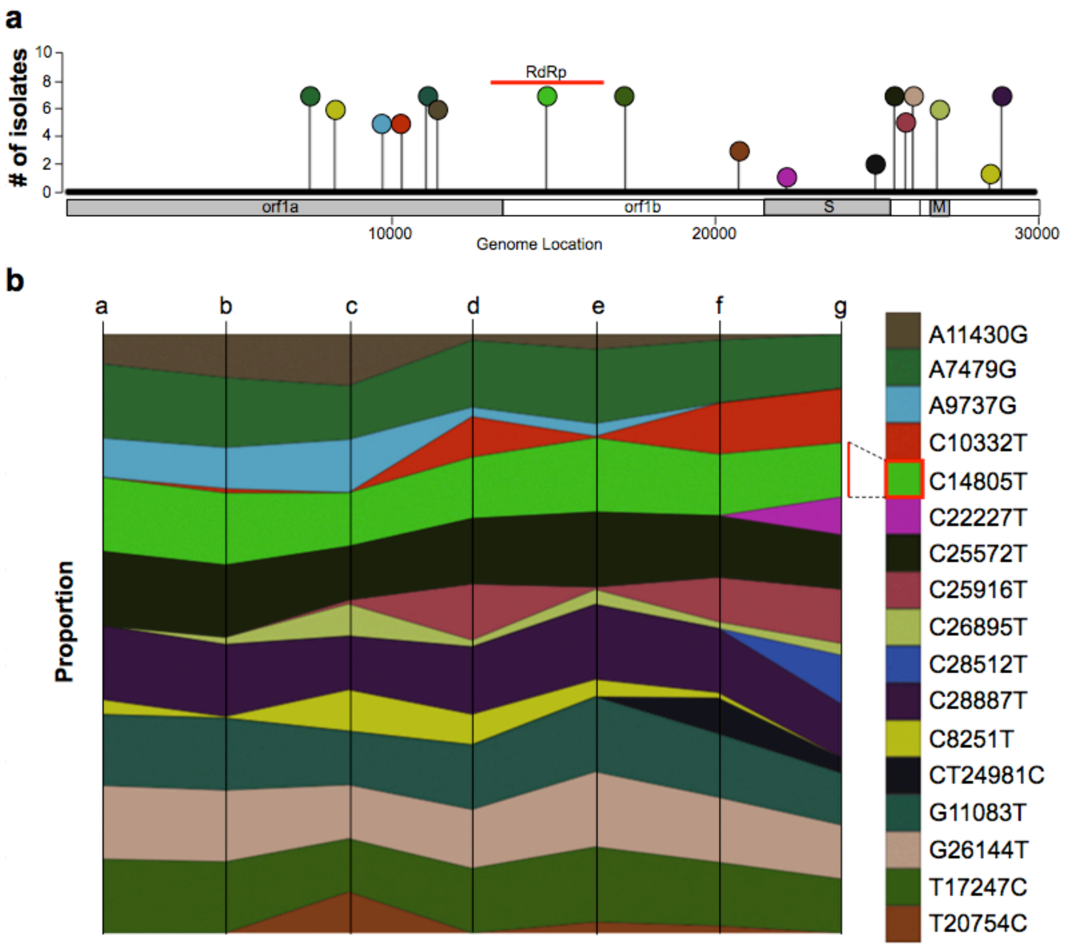

Supplementary Figure 2. (a) Location and frequency (number of isolates) of SNPs identified in isolates from Fig. 1a, coloured as in (b). (b) Relative abundances of SNPs present in isolates from Figure 1A. Height indicates the proportion of each variant as a fraction of all reads at that location, stacked so that the sum of proportions is the same for each isolate. The synonymous variant C14805T within the *nsp12* gene (encoding RdRp) is highlighted.

Supplementary Figure 3. Antigen specific CD8+ T cell responses to S1-peptide pools.

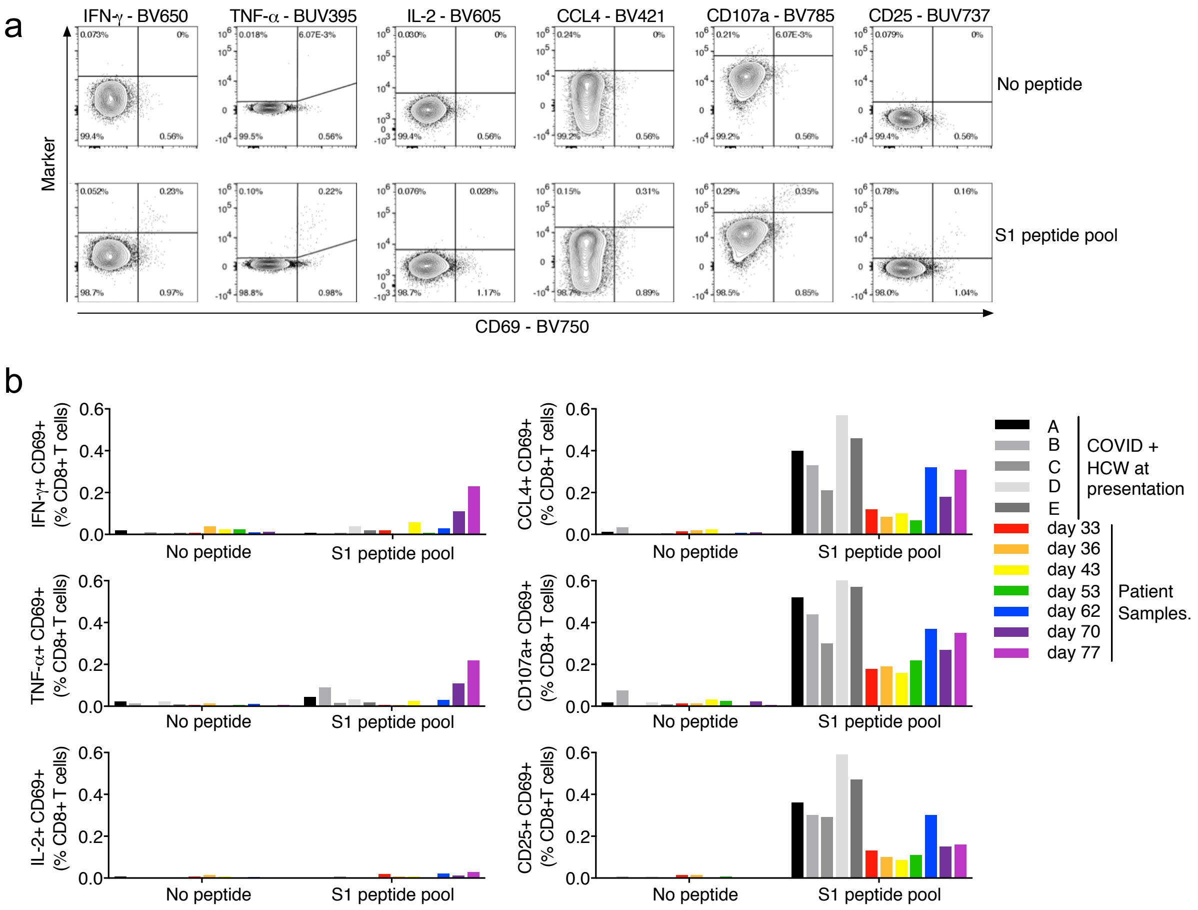

Figure S3. Representative flow cytometry data **(a)** and data for individual activation markers **(b)** from (Fig. 3a-b). For HCW, n=5 biologically independent subjects. Data from one independent experiment.

CITIID-NIHR COVID-19 BioResource Collaboration

Principal Investigators
Stephen Baker[1,2], John Bradley[3], Gordon Dougan[1,2], Ian Goodfellow[4], Ravi Gupta[1,2], Nathalie Kingston[3], Paul J. Lehner[1,2], Paul A. Lyons[1,2], Nicholas J. Matheson[1,2], Caroline Saunders[5], Kenneth G.C. Smith[1,2], M. Estee Torok[2,19], Mark Toshner[2], Michael P. Weekes[1,2]

NIHR Cambridge Clinical Research Facility Nurses, staff & volunteer research nurses
Anne Elmer[5], Carla Ribeiro[5], Jenny Kourampa[5], Sherly Jose[5], Giovanni Di Stephano[5], Karen Brookes[5], Isabel Cruz[5], Caroline McMahon[5], Vivien Mendoza[5], Jason Domingo[5], Gisele Alvio[5], Rnalie de Jesus[5], Ciro Pascuale[5], Areti Bermperi[5], Julie-Ann Zerrudo[5], Bensi Vergese[5], Marlyn Perales[5], Phoebe Vargas[5], Charmain Ocaya[5], Laura Watson[5], Katie Dempsey[5], Jieniean Worsley[5], Sharon Baker[5], Heather Jones[5], Jane Kennet[5], Jane Rowlands[5], Anne Meadows[5], Criona O'Brien[5], Rebecca Rastall[5], Cherry Crucusio[5], Sarah Hewitt[5], Jane Price[5], Jo Calder[5], Laura Canna[5], Ashlea Bucke[5], Hugo Tordesillas[5], Julie Harris[5], Valentina Ruffolo[5], Emily Li[5], Yvonne King[5]

Sample Logistics
Benjamin J Dunmore[2], Jennifer Martin[2], Ekaterina Legchenko[2], Carmen Treacy[2], Christopher Huang[2], Jennifer Wood[2], Rachel Sutcliffe[2], Josh Hodgson[2], Joy Shih[2], Stefan Graf[2], Zhen Tong[2], Federica Mescia[1,2], Tobias Tilly[2], Ciara O'Donnell[7], Kelvin Hunter[1,2], Linda Pointon[7], Nicole Pond[2], Marta Wylot[2], Emma Jones[2], Stuart Fawke[2], Ben Bullman[1], Maša Josipović[8]

Sample Processing
Laura Bergamaschi[1,2], Lori Turner[1,2], Isobel Jarvis[2], Ommar Omarjee[1,2], Aloka De Sa[1,2], Joe Marsden[2], Ariana Betancourt[1,2], Marianne Perera[2], Maddie Epping[1,2], Nathan Richoz[9], Georgie Bower[9], Rahul Sharma[2], Francesca Nice[10], Oisin Huhn[11], Stuart Fawke[2]

Sample acquisition
Lori Turner[1,2], Laura Bergamaschi[1,2], Natalia Savoinykh Yarkoni[1,2], Nika Romashova[2], Daniel Lewis[1,2], Andrew Hinch[1,2], Chiara Cossetti[12], Mateusz Strezlecki[13], Richard Grenfell[13], Oisin Huhn[11], Maddie Epping[1,2].

NIHR BioResource
John Allison[3], Heather Biggs[3], Helen Butcher[3], Daniela Caputo[3], Debbie Clapham-Riley[3], Eleanor Dewhurst[3], Christian Fernandez[3], Anita Furlong[3], Barbara Graves[3], Jennifer Gray[3], Tasmin Ivers[3], Emma Le Gresley[3], Rachel Linger[3], Mary Kasanicki[3], Sarah Meloy[3], Francesca Muldoon[3], Nigel Ovington[3], Sofia Papadia[3], Christopher J Penkett[3], Isabel Phelan[3], Venkatesh Ranganath[3], Jennifer Sambrook[3], Katherine Schon[3], Hannah Stark[3], Kathleen E Stirrups[3], Paul Townsend[3], Julie Von Ziegenweidt[3], Neil Walker[3], Jennifer Webster[3]

Papworth ICU
Ali Asaripour[14], Gary Polwarth[14], Alice Michael[14], Lucy Mwaura[14], Caroline Patterson[14]

Clinical Data Collection
Federica Mescia[1,2], Maša Josipović[8], Katherine Bunclark[15], Michael Mackay[15], Alice Michael[14], Sabrina Rossi[16], Mayurun Selvan[16], Sarah Spencer[17], Cissy Yong[16]

CUH ICU
Petra Polgarova[18], Charlotte Summers[2,18]

CUH Infectious Diseases Department
Effrossyni Gkrania-Klotas[19]

Cambridge COG Sequencing
M. Estee Torok[2,19], William Hamilton[2], Ian Goodfellow[4], Grant Hall[4], Aminu Jahun[4], Yasmin Chaudhry[4], Myra Hosmillo[4], Malte Pinckert[4], Illiana Georgana[4], Anna Takovleva[4], Laura Caller[4], Sarah Caddy[1], Teresa Feltwell[2], Fahad Khokhar[1], Luke Meredith[4], Charlotte Houldcroft[2], Martin Curran[20], Surendra Parmar[20]

1. Cambridge Institute of Therapeutic Immunology and Infectious Disease, Jeffrey Cheah Biomedical Centre, Cambridge Biomedical Campus, Cambridge, UK.
2. Department of Medicine, University of Cambridge School of Clinical Medicine, Cambridge Biomedical Campus, Cambridge, UK.
3. NIHR BioResource and NIHR Cambridge Biomedical Research Centre, Cambridge Biomedical Campus, Cambridge, UK.
4. Department of Pathology, University of Cambridge, Addenbrooke's Hospital, Cambridge, UK.
5. NIHR Cambridge Clinical Research Facility, Cambridge Biomedical Campus, Cambridge, UK.
6. Cambridge University Hospitals NHS Foundation Trust, Department of Microbiology, Cambridge, UK.
7. Department of Psychiatry, University of Cambridge, Cambridge, UK.
8. Department of Clinical Biochemistry, Institute of Metabolic Science, University of Cambridge, Cambridge, UK.
9. Molecular Immunity Unit, Medical Research Council Laboratory of Molecular Biology, University of Cambridge, Cambridge, UK.
10. Cancer Molecular Diagnostics Laboratory (CMDL), University of Cambridge, Cambridge, UK.
11. Department of Obstetrics & Gynaecology, University of Cambridge, Cambridge, UK.
12. Cambridge Institute for Medical Research, University of Cambridge, Cambridge, UK.
13. Cancer Research UK Cambridge Institute, Cambridge Biomedical Campus, Cambridge, UK.
14. Intensive Care Unit, Papworth Hospital, Cambridge, UK.
15. Papworth Hospital, Cambridge, UK.
16. Cambridge University Hospitals NHS Foundation Trust, Cambridge, UK.
17. Medical Research Council Toxicology Unit, University of Cambridge, Gleeson Building, Tennis Court Road, Cambridge, CB2 1QW, UK.
18. Intensive Care Unit, Cambridge University Hospitals NHS Foundation Trust, Cambridge, UK.
19. Infectious Diseases, Cambridge University Hospitals NHS Foundation Trust, Cambridge, UK.
20. Public Health England, Clinical Microbiology and Public Health Laboratory, Cambridge, UK.

MRC-Toxicology Unit COVID-19 Consortium

Anne Willis[1], Tuija Poyry[1], Angela Rubio Tenor[1], MariaVittoria Pizzinga[1], Robert Harvey[1], Gavin Garland[1], Marion MacFarlane[1], Nobu Morone[1], John Le Quesne[1], Leah Office[1], Ana Teodosio[1], Mie Monte[1], Kathryn Lilley[1], Michael Chapman[1], Emily Staples[1], Sarah Spencer[1], Tsz So[1], Tiffeney Mann[1], James E. D. Thaventhiran[1]

1. Medical Research Council Toxicology Unit, University of Cambridge, Gleeson Building, Tennis Court Road, Cambridge, CB2 1QW, UK.
