# Supplementary material for: Treatment of COVID-19 with remdesivir in the absence of humoral immunity: a case report
Source: Nat Commun. 2020 Dec 14;11:6385. doi: 10.1038/s41467-020-19761-2 (PMC7736571; doi:10.1038/s41467-020-19761-2)
Supplement: Supplementary file 2 — Reporting Summary [file 41467_2020_19761_MOESM2_ESM.pdf]

nature research

Corresponding author(s): Matthew S. Buckland, Nick M. Matheson, James Thaventhiran

Last updated by author(s): 2020-10-9

Reporting Summary

Nature Research wishes to improve the reproducibility of the work that we publish. This form provides structure for consistency and transparency in reporting. For further information on Nature Research policies, see our Editorial Policies and the Editorial Policy Checklist.

Statistics

For all statistical analyses, confirm that the following items are present in the figure legend, table legend, main text, or Methods section.

n/a	Confirmed	
☐	☒	The exact sample size (*n*) for each experimental group/condition, given as a discrete number and unit of measurement
☒	☐	A statement on whether measurements were taken from distinct samples or whether the same sample was measured repeatedly
☒	☐	The statistical test(s) used AND whether they are one- or two-sided<br>*Only common tests should be described solely by name; describe more complex techniques in the Methods section.*
☒	☐	A description of all covariates tested
☒	☐	A description of any assumptions or corrections, such as tests of normality and adjustment for multiple comparisons
☐	☒	A full description of the statistical parameters including central tendency (e.g. means) or other basic estimates (e.g. regression coefficient) AND variation (e.g. standard deviation) or associated estimates of uncertainty (e.g. confidence intervals)
☒	☐	For null hypothesis testing, the test statistic (e.g. *F*, *t*, *r*) with confidence intervals, effect sizes, degrees of freedom and *P* value noted<br>*Give P values as exact values whenever suitable.*
☒	☐	For Bayesian analysis, information on the choice of priors and Markov chain Monte Carlo settings
☒	☐	For hierarchical and complex designs, identification of the appropriate level for tests and full reporting of outcomes
☒	☐	Estimates of effect sizes (e.g. Cohen's *d*, Pearson's *r*), indicating how they were calculated

Our web collection on statistics for biologists contains articles on many of the points above.

Software and code

Policy information about availability of computer code

Data collection	Viral isolates were analysed by Nanopore sequencing as part of the COG-UK sequencing project, following the ARTICnetwork V3 protocol (https://dx.doi.org/10.17504/protocols.io.bbmuik6w), and assembled using the ARTICnetwork assembly pipeline (https://artic.network/ncov-2019/ncov2019-bioinformatics-sop.html, version 1.1.0). Cytek Aurora cytometry data was collected using SpectroFlo v2.2. FACS data was analysed using flowjo v10.6.2. Luminex data was analysed using Exponent Software V31.
Data analysis	Variants were initially assessed using the ARTICnetwork assembly pipeline VCF output files, with a SNP being called when >50.1% frequency at a single base. These were then independently confirmed using iVar analysis (https://andersen-lab.github.io/ivar/html), set to a quality threshold of Q20 and a minimum frequency of 10%, with a SNP being called when >50.1% of the reads at a particular position differed from the reference sequence, Wuhan-Hu-1.

For manuscripts utilizing custom algorithms or software that are central to the research but not yet described in published literature, software must be made available to editors and reviewers. We strongly encourage code deposition in a community repository (e.g. GitHub). See the Nature Research guidelines for submitting code & software for further information.

Data

Policy information about availability of data

All manuscripts must include a data availability statement. This statement should provide the following information, where applicable:

- Accession codes, unique identifiers, or web links for publicly available datasets
- A list of figures that have associated raw data
- A description of any restrictions on data availability

Data Availability :Viral sequence data, that was compared to GenBank Accession MN908947.3 (Wuhan-Hu-1) is available from COVID-19 Genomics UK and the GSAID. The accession numbers are detailed in Extended Data Table 5.

Field-specific reporting

Please select the one below that is the best fit for your research. If you are not sure, read the appropriate sections before making your selection.

- [x] Life sciences
- [ ] Behavioural & social sciences
- [ ] Ecological, evolutionary & environmental sciences

For a reference copy of the document with all sections, see nature.com/documents/nr-reporting-summary-flat.pdf

Life sciences study design

All studies must disclose on these points even when the disclosure is negative.

Sample size	This is a case report in a single individual, compared to available control samples. The conclusions drawn from these comparisons are appropriate considering the variance within the control samples.
Data exclusions	No data has been excluded from analysis.
Replication	Replication was not applicable as this is an observational study of the response to drug challenge and rechallenge in a single individual infected with COVID-19.
Randomization	Randomization was not applicable as this is an observational study of the response to drug challenge and rechallenge in a single individual infected with COVID-19.
Blinding	Blinding was not applicable as this is an observational study of the response to drug challenge and rechallenge in a single individual infected with COVID-19.

Reporting for specific materials, systems and methods

We require information from authors about some types of materials, experimental systems and methods used in many studies. Here, indicate whether each material, system or method listed is relevant to your study. If you are not sure if a list item applies to your research, read the appropriate section before selecting a response.

Materials & experimental systems

n/a	Involved in the study	
[ ]	[x]	Antibodies
[ ]	[x]	Eukaryotic cell lines
[x]	[ ]	Palaeontology and archaeology
[x]	[ ]	Animals and other organisms
[ ]	[x]	Human research participants
[x]	[ ]	Clinical data
[x]	[ ]	Dual use research of concern

Methods

n/a	Involved in the study	
[x]	[ ]	ChIP-seq
[ ]	[x]	Flow cytometry
[x]	[ ]	MRI-based neuroimaging

Antibodies

Antibodies used

TCC; capture antibody; Clone Name: aE11; Hycult Biotech HM2167-100UG; Lot number: 28954M0420.
anti-CD107a BV785; Clone Name: H4A3; Biolegend 328644;
anti-CD28; Clone Name: CD28.2; ThermoFisher 16-0289-85;
anti-CD49d: Clone Name: R1-2; ThermoFisher 16-0499-85;
anti-CD3-FITC: Clone Name: UCHT1; Biolegend 300406;
anti-CD4-AF700: Clone Name: RPA-T4; BD Biosciences 557922;
anti-CD8-PECY7: Clone Name: RPA-T8; Biolegend 301012.

Validation

TCC; capture antibody, clone aE11: This antibody has been validated by the manufacturer for use on human samples for functional blockade of activity. Validation statement by manufacturer ,"Antibody aE11 inhibits platelet activation by antiphospholipid antibody serum." Citation: Stewart, M et al. Antiphospholipid antibody-dependent C5b-9 formation. BJH 1997, 96:451 6. Meuwissen, M et al. Colocalisation of intraplauq C reactive protein, complement, oxidised low density lipoprotein, and macrophages in stable and unstable angina and acute myocardial infarction. J Clin Path 2005, 59:126

Anti-CD107a clone H4A3: The antibody has been validated by the manufacturer for use FACS use on human samples: Their validation statement, "This antibody is specific to human LAMP-1. Positive control: Hela cells; LAMP-1 molecular weight appears to be at ~110 kDa on the gel due to high glycosylation." Product Citations:

Vargas-Inchaustegui D, et al. 2017. J Immunol. 10.4049/jimmunol.1700586. PubMed
Niessl J, et al. 2020. EBioMedicine. 54:102727. PubMed

anti-CD28 clone CD28.2: The antibody has been validated by the manufacturer for FACS use on human samples: Their validation statement, "TheCD28.2 antibody has been tested by flow cytometric analysis of normal human peripheral blood cells. This can be used at less than or equal to 1 µg per test. A test is defined as the amount (µg) of antibody that will stain a cell sample in a final volume of 100 µL. Cell number should be determined empirically but can range from 10^5 to 10^8 cells/test."
Citations: Journal of immunology research "Tumor-activated TCRγδ⁺ T cells from gastric cancer patients induce the antitumor immune response of TCRαβ⁺ T cells via their antigen-presenting cell-like effects." Mao C,Mou X,Zhou Y,Yuan G,Xu C,Liu H,Zheng T,Tong J,Wang S,Chen D 2015 .

anti-CD49d: clone R1-2: The antibody has been validated by the manufacturer for FACS use on human samples: Their validation statement, "The 9F10 antibody has been tested by flow cytometric analysis of normal human peripheral blood cells. This can be used at less than or equal to 1 µg per test." Citations: Virology, "Impact of antibody quality and anamnestic response on viremia control post-challenge in a combined Tat/Env vaccine regimen in rhesus macaques." Authors Demberg T,Brocca-Cofano E,Kuate S,Aladi S,Vargas-Inchaustegui DA,Venzon D,Kalisz I,Kalyanaraman VS,Lee EM,Pal R,DiPasquale J,Ruprecht RM,Montefiori DC,Srivastava I,Barnett SW,Robert-Guroff M 2013

Anti-CD3-FITC clone UCHT1: The antibody has been validated by the manufacturer for FACS use on human samples: Their validation statement, "Each lot of this antibody is quality control tested by immunofluorescent staining with flow cytometric analysis."
Citations: Thakral D, et al. 2008. J. Immunol. 180:7431.

Anti-CD4-AF700 clone RPA-T4: The antibody has been validated by the manufacturer for FACS use on human samples: Their validation statement, "This antibody is routinely tested by flow cytometric analysis." Citations: Schlossman SF, Boumsell L, Gilks W, et al, ed. Leukocyte Typing V: White Cell Differentiation Antigens. New York: Oxford University Press; 1995. (Clone-specific)

Anti-CD8-PECY7 clone RPA-T8: The antibody has been validated by the manufacturer for FACS use on human samples: Their validation statement, "Each lot of this antibody is quality control tested by immunofluorescent staining with flow cytometric analysis." Product citation, Stoeckius M, et al. 2017. Nat. Methods. 14:865..

Eukaryotic cell lines

Policy information about cell lines

Cell line source(s)	HEK 293T cells were obtained from Lehner laboratory stocks the original commercial supplier is unknown. This line has been authenticated by STR profiling.(See below).
Authentication	STR profiling (Menzies et al., 2018: PMID: 30543180; Miles et al., 2017 PMID: 28296633)
Mycoplasma contamination	Mycoplasma contamination: Cells were confirmed to be Mycoplasma negative (MycoAlert, Lonza)
Commonly misidentified lines (See ICLAC register)	No commonly misidentified cell lines were used in the study.

Human research participants

Policy information about studies involving human research participants

Population characteristics	The subject of the study is a male aged 31 with X-linked agammaglobulinaemia. This is treated with life-long replacement immunoglobulin infusions.
Recruitment	The subject was recruited into a bioresource study for monitoring the clinical course of COVID-19. This single-case report was undertaken due to the unique genotype of the subject within the cohort and their distinctive response to treatment. There is no bias in a report of this nature, but as a single case-report we provide no data in regard to how a diverse human population behaves in response to COVID-19 challenge or remdesivir treatment.
Ethics oversight	The study was approved by the East of England – Cambridge South national institutional ethics review board (17/EE/0025). The patient provided written informed consent. Additional healthy controls, patients and healthcare workers with COVID-19 were enrolled to the NIHR BioResource Centre Cambridge (17/EE/0025) and the Oxford Gastrointestinal Illness Biobank (16/YH/0247).

Note that full information on the approval of the study protocol must also be provided in the manuscript.

Flow Cytometry

Plots

Confirm that:

- [x] The axis labels state the marker and fluorochrome used (e.g. CD4-FITC).
- [x] The axis scales are clearly visible. Include numbers along axes only for bottom left plot of group (a 'group' is an analysis of identical markers).
- [x] All plots are contour plots with outliers or pseudocolor plots.
- [x] A numerical value for number of cells or percentage (with statistics) is provided.

Methodology

Sample preparation	Intracellular cytokine staining: Surface staining was performed at 4 C for 30 mins. Cells were then washed two times in FACS buffer, and fixed and permeabilized at 4 C for 30 mins using BD Cytofix/Cytoperm solution. Cells were then washed twice with 1x BD Perm/Wash buffer, and stained for intracellular markers at 4C for 30 mins. Two further washes with 1x BD Perm/Wash buffer were performed and cells were stored in FACS buffer at 4C.<br>Cultured cells were washed with FACS wash buffer (Biolegend)
Instrument	For ICS analysis Cytek Aurora spectral analyzer (4 laser; UV, violet, blue, and red). For proliferation assessment BD LSR II.
Software	Aurora data was gathered with SpectroFlo v2.2. Data were analyzed using flowjo v10.6.2.
Cell population abundance	All gated populations make up at least 10% of the live cells within the population.
Gating strategy	Gates for intracellular cytokine staining were set by the flourescence characteristics of unstimluated control cells.

- [x] Tick this box to confirm that a figure exemplifying the gating strategy is provided in the Supplementary Information.
